# EEG-Microstates Reflect Auditory Distraction After Attentive Audiovisual Perception Recruitment of Cognitive Control Networks

**DOI:** 10.3389/fnsys.2021.751226

**Published:** 2021-12-09

**Authors:** Ute Korn, Marina Krylova, Kilian L. Heck, Florian B. Häußinger, Robert S. Stark, Sarah Alizadeh, Hamidreza Jamalabadi, Martin Walter, Ralf A. W. Galuske, Matthias H. J. Munk

**Affiliations:** ^1^Systems Neurophysiology, Department of Biology, Darmstadt University of Technology, Darmstadt, Germany; ^2^Department of Psychiatry and Psychotherapy, University of Tuebingen, Tuebingen, Germany; ^3^Department of Psychiatry and Psychotherapy, Jena University Hospital, Jena, Germany; ^4^NTT DATA Deutschland GmbH, Munich, Germany; ^5^Department of Psychiatry and Psychotherapy, Philipps-University, Marburg, Germany

**Keywords:** electroencephalography, microstates, audiovisual, crossmodal, attention, distraction, resting states, background music

## Abstract

Processing of sensory information is embedded into ongoing neural processes which contribute to brain states. Electroencephalographic microstates are semi-stable short-lived power distributions which have been associated with subsystem activity such as auditory, visual and attention networks. Here we explore changes in electrical brain states in response to an audiovisual perception and memorization task under conditions of auditory distraction. We discovered changes in brain microstates reflecting a weakening of states representing activity of the auditory system and strengthening of salience networks, supporting the idea that salience networks are active after audiovisual encoding and during memorization to protect memories and concentrate on upcoming behavioural response.

## 1. Introduction

Background noise is a ubiquitous phenomenon which increasingly infiltrates processes of our daily life, e.g., background music in supermarkets, street noise and traffic noise. It can also be found in current media, e.g., by unwanted advertisements, but also when watching movies and during reports and news, when attention should be focused maximally on relevant information and complex processes. In order to elucidate the effects of background noise several studies investigated students' performance and attention while doing their homework when simultaneously distracting sounds in the background were presented. It was shown that these inhibited their performance depending on the kind of background noise (Furnham and Bradley, 1997; Pool et al., 2000).

However, the effects of sensory distraction have barely been investigated with respect to their influence on brain states supporting multimodal perception and working memory. To date, much is known about cross-modal interference during stimulus processing (Spence et al., 2000; Calvert, 2001; Weissman et al., 2004), but hardly any data are available on the dynamics of brain states before and after encoding of simultaneously perceived information in different sensory modalities. In particular, the respective resting state activity might be of fundamental importance. Generally speaking, resting state (RS) activity in the electroencephalography (EEG) can be described as a short time period of semi-stable electric field topographies, called microstates (MS) (Lehmann et al., 1987; Lehmann, 1990). These small numbers of topographies are stable for tens of milliseconds and transition into another semi-stable pattern (Koenig et al., 2002). General data analysis approaches of MS consider duration, occurrence, contribution and transition rate in order to describe MS topographies and commonly at least four different MS topographies can be differentiated (Koenig et al., 2002; Michel and Koenig, 2018). These MS are labelled with letters and each letter reflects one topography: MS A resembles a left frontal and to right occipito-temporal dipole, MS B resembles a right frontal and to left occipito-temporal dipol, MS C resembles a anterior-posterior dipol and MS D resembles a fronto-central and occipital-temporal dipol. Some studies have established relations between MS dynamics and various RS brain networks as revealed by functional magnetic resonance imaging (fMRI) (Britz et al., 2010) or in electrophysiological RS networks (Custo et al., 2017, for Review see Michel and Koenig, 2018).

Moreover, Weissman et al. (2004) investigated cross-modal interference between visual and auditory stimuli using fMRI and found that the sensory cortices play novel roles in increasing attention to goal-relevant stimuli. More precisely, the dorsolateral prefrontal cortex (DLPFC) and the anterior cingulate cortex (ACC) are involved in increasing attention when distracting auditory stimuli conflict with visual stimulation (Weissman et al., 2004). Seeley et al. (2007) identified the salience network, which is anchored in dorsal anterior cingulate and orbital frontoinsular cortices (Seeley et al., 2007). This network is being involved in controlling and coordinating domains of cognitive control, e.g., attention, working memory, inhibition and planning (Seeley et al., 2007; Niendam et al., 2012; Breukelaar et al., 2017). Britz et al. (2010) found that MS C is associated with the salience network (Britz et al., 2010). Since certain MS reflect network activity in the brain areas found by Weissman et al. (2004), e.g., ACC and DLPFC, it is most promising that we choose MS analysis as evaluation method for the RS recordings in our cross-modal task (Weissman et al., 2004).

**Figure 1:**
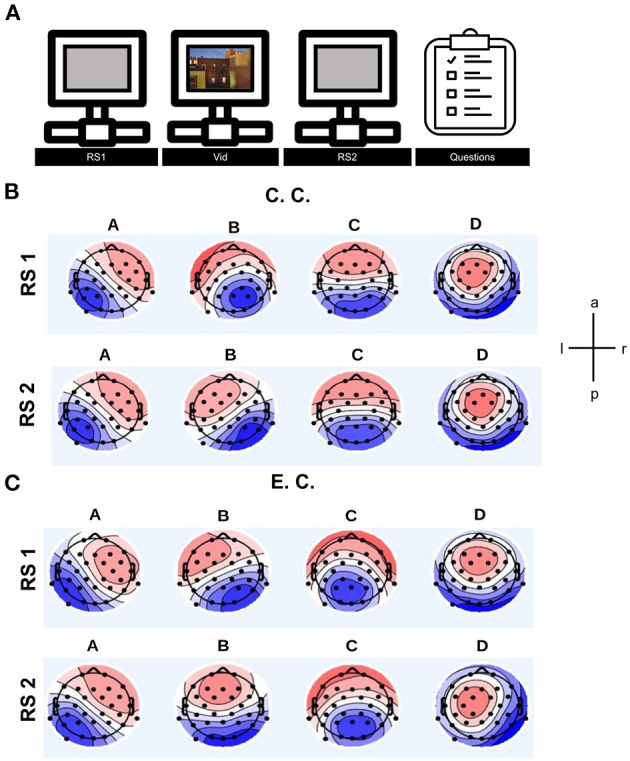
**(A)** Schematic illustration of the experimental task showing duration of resting state measurements, playing video and questionnaire. **(B)** Averaged microstate topographies in the control condition (C.C.) for both resting state measurements. **(C)** Averaged microstate topographies in the experimental condition (E.C.) for both resting state measurements. After clustering, the individual and group MS maps were reordered with reference to a template map. For the template map an average of all individual MS patterns from all subjects including all experimental conditions from both resting states recordings was taken. Comparing the topographies of MS B in both resting states in control condition, there is a difference in the topography. In the experimental condition (auditory distraction) is a small difference in the topography of MS A, MS B and MS C. Upper and lower rows provide MS topographies of the resting state measurement before and after watching the video, respectively. Topographic maps of opposite polarity are randomly coded in red and blue using a linear color scale. Polarity is ignored by MS analysis. Left ear is shown on the left, with the nose pointing upwards. The four topographies cover large areas of the scalp and represent global electrical brain processes. RS1, resting state before showing the video; RS2, resting state after showing the video.

We wanted to investigate the influence of auditory interference in an audio-visual, attention-demanding paradigm to get a sense of the day-to-day environmental influences on the brain. Therefore, we investigated the effect of distraction by auditory background noise rather than visual noise interfering with auditory information. For this purpose, we developed a paradigm in which subjects were instructed to watch a video and memorise as many auditory and visual items as possible in order to recall content later in a questionnaire, not knowing that the effect of auditory distraction would be the target of the project. To this end, we prepared an audiovisual documentation which was presented to the subjects with or without background “noise” under varying levels of impact (experimental condition: auditory distraction, control condition: without auditory distraction) while measuring brain states from EEG recordings (Figure 1A). MS analyses were applied to two RS measurements, one recorded before and one after video presentation.

Based on previous work (Weissman et al., 2004), we propose that auditory distraction by loud background noise should modify ongoing brain activity captured in RS recordings after active perception and encoding of the audio-visual task. Topographically, two MS are located frontally and occipitally, but laterally shifted [clockwise (MS A)/counterclockwise (MS B)]. Both include parts of the visual and auditory cortex. For this reason, we assume that our audio-visual paradigm with strong auditory background distraction has an impact on these two MSs.

Furthermore, we predict an increase in microstate parameters (contribution, occurrence and duration) and their transition rates in the experimental condition (with auditory distraction) of MS C, which seems to reflect the salience network or parts of the intrinsic default mode network (Britz et al., 2010; Custo et al., 2017; Michel and Koenig, 2018). Topographically, MS C shows opposite patterns between frontoparietal and occipital regions, which include the visual cortex, DLPFC, posterior cingulate cortex (PCC), precuneus and ACC (Britz et al., 2010; Custo et al., 2017). Regarding the behavioural test results, we propose that auditory background distraction would have a negative impact on subjects' recall performance (Furnham and Bradley, 1997; Pool et al., 2000).

## 2. Methods

### 2.1. Paradigm

The background-music paradigm was developed to test the influence of auditory distraction on brain electrical states. Subjects were shown a TV feature about global inequality (poverty, wealth gap) of 5 min duration. The control group (C.C.) watched the video with informative and non-vocal, decent background sounds, while the experimental group (E.C.) watched the video with informative sound and mostly vocal, loud and popular music (however, the lyrics mostly fitted well with current information context). We considered this musical background as distracting. Subjects were instructed at the beginning of the experiment to attentively watch and listen, as they would subsequently have to answer questions about the content. The RS recordings (5 min) were taken just before and immediately after showing the video (Figure 1A). During RS recording, subjects fixed their gaze at the centre of a grey screen with eyes open. Subjects were assigned to the groups (C.C. and E.C.) in a pseudo-randomised manner.

Subjects were asked eight questions about the content of the video (Questionaire is available in Supplementary Section 1.1). Here, 6 binary items and 2 multinomial items were used. For subsequent data analysis operations, these multinomial variables were binarized (dummy-coding) for ensuring comparability. One questionnaire from the experimental group with auditory distraction was lost, only 13 questionnaires could be evaluated for this group.

### 2.2. Subjects

30 subjects volunteered to perform our experiment. Written informed consent was obtained from all participants. After pre-processing, data sets from 13 subjects who performed the control condition and from 14 subjects who performed the experimental condition qualified for further analysis. The age range of all subjects was 22–33 years (mean = 25, SD = 3.1, median = 24). In the control condition 5 male and 8 female subjects and in the experimental condition 7 male and 7 female subjects were measured.

### 2.3. Registering Brain Activity

30 EEG-electrodes (actiCap, Brain Products GmbH, Germany) were placed in standard positions (10-20-system) on the head (see Supplementary Figure S1). Electrode impedance was improved until ≤5 kΩ. Data were sampled with 250 or 500 Hz.

### 2.4. EEG Pre-processing

Pre-processing functions of the MATLAB toolbox EEGLAB (v. 2019_1) were used to cope with known signal components related to biological / technical noise, e.g., eye blinks and line noise (Delorme and Makeig, 2004). Each dataset was first bandpass filtered between 0.3 and 200 Hz and subsequently segmented into epochs of 1 s duration. Also, muscle and motion artefacts as well as open (>120 μV) and flat channels (<5 μV) were removed.

Furthermore, independent component analysis (ICA) was employed to extract signal components reflecting eye movement and continuous muscle activity (Cohen, 2014). Components can be interpreted based on their topographies, time courses and frequency spectra. Components which contain flat dynamics, sporadic high-amplitude spikes and were spatially located in anterior brain regions were removed as eye blinking. Whereas, components which contain bursts between 20 to 40 Hz activity and with a high amplitude, located near the face, neck or ears were removed as muscle activity (Cohen, 2014).

### 2.5. Microstate Analysis

The EEGLAB plugin for microstates (v1.1) by Thomas Koenig ^1^ was used for MS analysis. Pre-processed EEG data were further bandpass-filtered between 2 and 20 Hz and re-referenced to average reference. Due to the fact that in some experiments sampling rates were 500 Hz and in others 250 Hz, we reduced sampling rate for all data sets to 250 Hz to ensure comparability. The entire dataset consisted of recordings with one or the other experimental/control condition (C.C./E.C.) and two RS recordings (RS1/RS2) per subject. We analysed exactly 5 min of both RS recordings, as this was the smallest common recording duration in all subjects.

In accordance with Murray et al. (2008), we calculated global field power peaks (GFP) for each epoch, which constitutes a reference-independent measure of response strength (Murray et al., 2008). Subsequently, we followed the methodology of Krylova et al. (2021) for MS analysis, for more details see Supplementary Section 1.2 (Krylova et al., 2021).

We derived the following dynamic parameters for each MS providing averaged (arithmetic mean) values:

- duration (period in which consecutive maps were assigned to the same MS class),
- occurrence (mean number of MS per second),
- contribution (percentage time occupied in each MS) and finally
- transition rate (original/delta) (Koenig et al., 2002).

Contribution is a percentage representation of the MSs over the period of the recording duration. The transition rate between all MSs was expressed as probability with respect to all possible transitions (original transition rate). The original transition rate was then related to the theoretical transition model of Lehmann et al. (2005), the latter providing the observed deviation from equal transition probability for all possible transitions (delta transition rate) (Lehmann et al., 2005).

### 2.6. Statistical Analysis

Statistical analysis was performed in MATLAB [version: 9.5.0.1178774, (2018b), Natick, Massachusetts: The MathWorks Inc.].

To test for equal medians between C.C. and E.C., the two-sided Wilcoxon rank sum test was used (function *ranksum*), which is very close to the Mann-Whitney U test (MWU). Wilcoxon signed rank tests (function *signrank*) were conducted for testing RS1 and RS2 within each separate experimental condition (Hollander and Douglas, 1999; Gibbons and Chakraborti, 2010). For further details see Supplementary Section 1.3.

The Bonferroni Correction was used to adjust for multiple comparisons of the MS parameters duration, occurrence and contribution, whereas the False Discovery Rate (FDR) was used for the delta and original transition rate of MSs (Genovese et al., 2002; Cohen, 2014; Fachada and C. Rosa, 2018).

For the evaluation of the questionnaires, we used non-parametric statistical methods (Mann-Whitney U test, χ^2^-test) for assessing the differences of central tendencies between control and experimental groups.

## 3. Results

We analysed 30-channel resting state EEG-recordings of 27 subjects before and after they memorised visual and auditory objects which they were asked for in a questionnaire. Both groups (C.C. *n* = 13/ E.C. *n* = 14) performed indistinguishably well, on average recalling 8 items (C.C.: 8.2; E.C.: 7.6), the two groups differed only by a non-significant trend (χ^2^-test: *p* = 0.77; MWU-test: tied-p = 0.49) that distracted observers tended to memorise less well.

After clustering and reordering steps, the final topographies of all MSs were estimated. The averaged topographies per condition and resting state recording were analysed (Figures 1B,C). Further description and discussion about the topographies is available in the Supplementary Sections 1.4 and 1.5.

### 3.1. MS Parameters and Transition Rates of the C.C.

**Figure 2:**
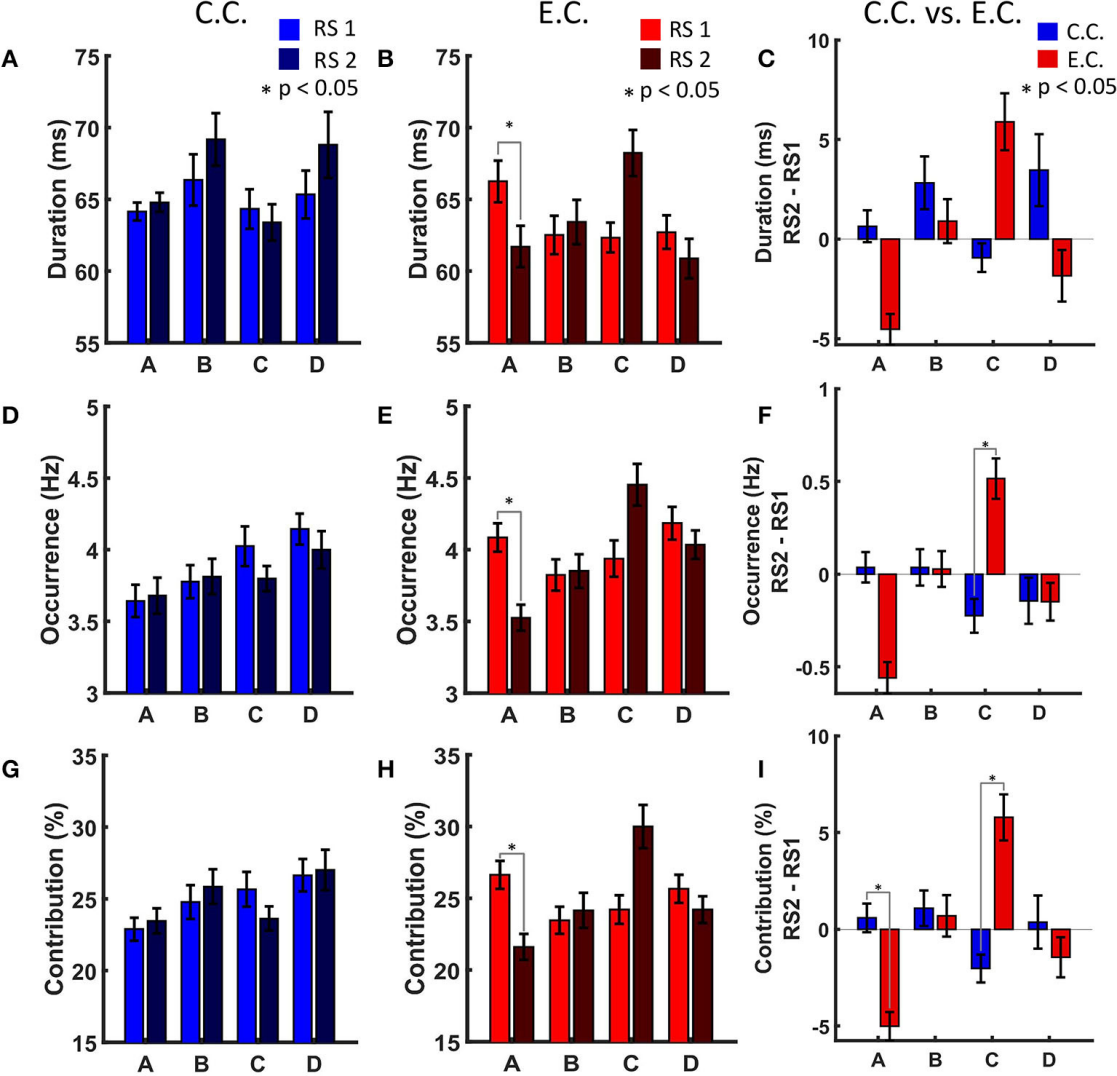
MS parameters duration, occurrence and contribution. **(A)** MS parameter duration in the control condition. **(B)** MS parameter duration in the experimental condition with auditory distraction. MS A shows a significant decrease (*p* < 0.05) in RS2. **(C)** MS parameter duration in comparison of both experimental conditions. Light colour indicates resting state recording before showing the video. Dark colour indicates resting state recording after showing the video. **(D)** MS parameter occurrence in the control condition. **(E)** MS parameter occurrence in the experimental condition. MS A shows a significant decrease (*p* < 0.05) in RS2. **(F)** MS parameter occurrence in comparison of both experimental conditions. MS C shows a significant increase (*p* < 0.05) after auditory distraction. **(G)** MS parameter contribution in the control condition. **(H)** MS parameter contribution in the experimental condition. MS A shows a significant decrease (*p* < 0.05) in RS2. **(I)** MS parameter contribution in comparison of both experimental conditions. MS A shows a significant decrease (*p* < 0.05) in RS2. MS C shows a significant increase (*p* < 0.05) after auditory distraction. Fourteen Subjects were analysed for the experimental condition and 13 Subjects for the control condition. The results of the RS recordings were subtracted from each other to obtain a RS independent comparison between the two conditions. Blue, control condition (C.C.); red, experimental condition (E.C.); x-axis, MS classes; y-axis, each MS parameter. Error bars indicate standard error of the mean (* indicates *p* < 0.05).

**Figure 3:**
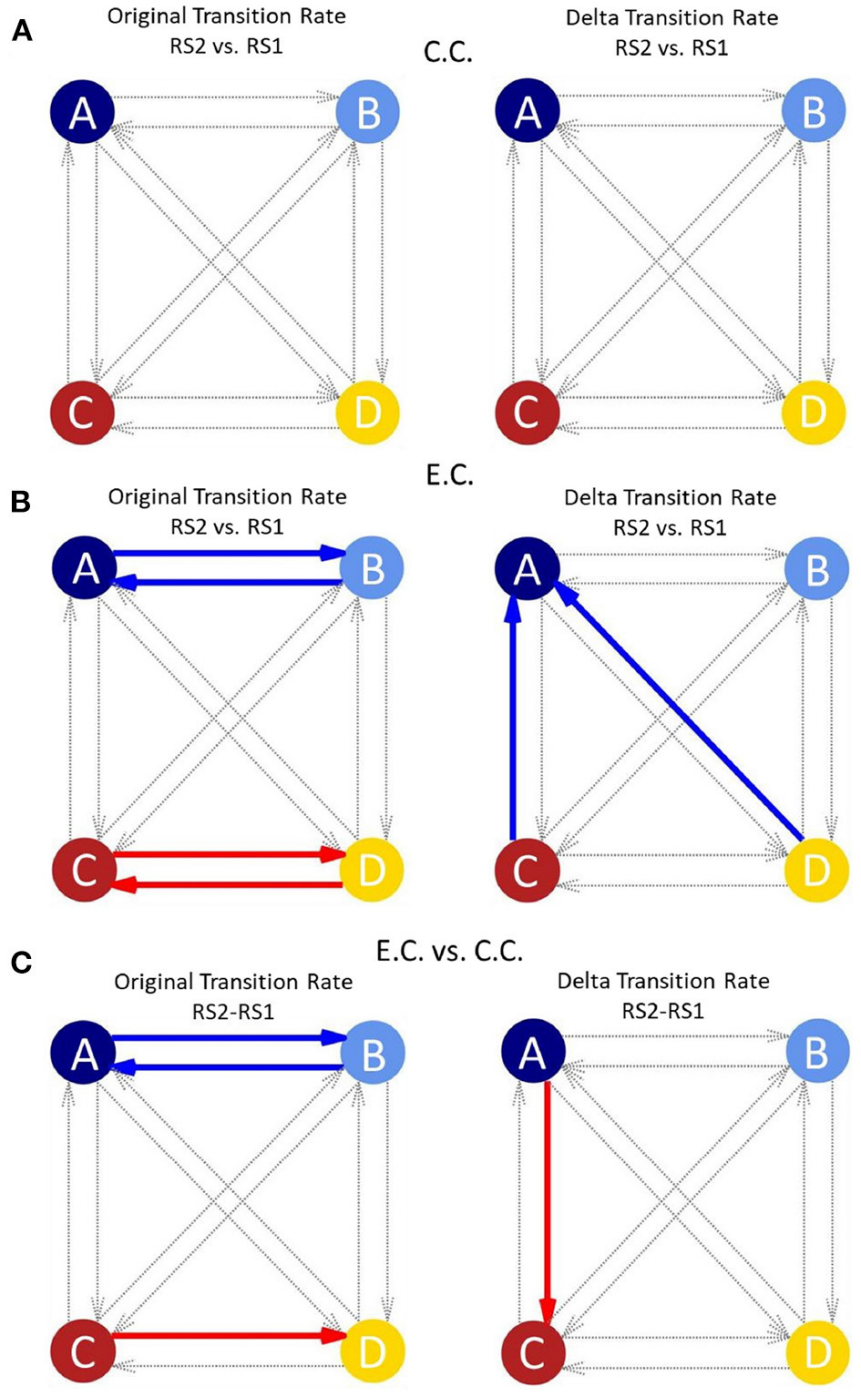
**(A)** Network graphs of the transition rate (original/delta) of the four canonical MSs for the control condition comparing the two resting state measurements (modified from Krylova et al., 2021). In the control condition, no significant results could be obtained for any of the transition rates between MSs (thin grey arrows). The original transition rate is influenced by the occurrence of MSs, whereas, the delta transition rate is independent of the occurrence of MSs. **(B)** Network graphs of the transition rate (original/delta) of the four canonical MSs for the experimental condition (with auditory distraction) comparing the two resting state measurements. Clearly differences between the original and delta transition rate are visible. Original transition rate shows significant shifts (coloured thick arrows) between MS A and MS B and between MS C and MS D. The delta transition rate shows significant shifts from MS C and MS D to MS A. **(C)** Network graphs of the transition rate (original/delta) in comparison of the experimental and control condition and the resting state recordings. There are differences for the transitions between the original and delta transition rates. The original transition rate shows significant shifts between MS A and MS B and from MS C to MS D. Whereas the delta transition rate only shows a significant shift from MS A to MS C. Each circle indicates one MS (A–D); red arrows: increased transition rate; blue arrows: decreased transition rate; thick arrows: *p*-value between 0.05 and 0.01; dotted grey arrows: transitions between MSs with p > 0.05; E.C., experimental condition; C.C.: control condition; RS1, resting state before showing the video; RS2, resting state after showing the video; MS, Microstate.

We could hardly see any significant changes in MS parameters for the control group after watching and memorising the video in comparison of the two RS recordings (Figures 2A,D,G). No significant results were also observed for MS transitions in the control condition (Figure 3A, as we have already illustrated in Krylova et al., 2021).

### 3.2. MS Parameters and Transition Rates of the E.C.

We found that distracting music after active audiovisual perception had a significant impact on all MS parameters of two of the four investigated MSs. Statistical analysis revealed that these effects occurred both for MS A and MS C. MS A showed a significant decrease for the parameter's duration, occurrence and contribution during RS2 as compared to RS1 for the E.C. (Figures 2B,E,H).

The relative frequencies of transitions between different MSs provide information on the overall timing of brain states and preponderance of networks: here, MS A, MS C and MS D showed significant changes in transition rate, which indicates the timing of the MSs (Figure 3B). This allows for statements to be made about which MS occurs more often and whether a transition to a specific MS is preferred. As displayed in Figure 3B on the left, the original transition rate shows significant results for the transitions between MS A and MS B and for the transitions between MS C and MS D during RS2 as compared to RS1 for the E.C.

Transitions from MS C to MS A and from MS D to MS A show significant results for the delta transition rate during RS2 as compared to RS1 in the E.C. (Figure 3B on the right). The delta transition rate is estimated as the difference between the observed and the theoretical transition rate (Lehmann et al., 2005).

### 3.3. MS Parameters and Transition Rates in Comparison Between C.C. and E.C.

Comparing E.C. and C.C. (with RS2 minus RS1) auditory distraction influences MS C more than MS A (Figures 2C,F,I). Contribution of MS A shows a significant decrease (Figure 2I), while occurrence and contribution of MS C show significant increases for the condition with background music distraction (Figures 2F,I). For the original transition rate we found significant decreases for the transitions between MS A and MS B (in both directions) and a significant increase for the transitions between MS C to MS D (also in both directions in Figure 3C on the left). The delta transition rate shows a significant increase for the transitions from MS A to MS C (Figure 3C on the right).

## 4. Discussion

Distracting auditory stimuli impact MSs occurrence and contribution during RS2 while keeping visual and auditory items in memory. To our surprise, MS A occurred less frequently and was shorter when distracting music interfered during encoding, while MS C increased in duration and occurrence. It was not expected that the audiovisual paradigm had no impact on the parameters of MS B.

### 4.1. Cross-Modal Interaction Effects on MS A

In MS A, the topography is slightly shifted and covers upon distraction more temporal areas, as shown in the red- and blue-coloured patterns in Figures 1B,C. The primary areas of the visual system and the auditory system are located in these regions.

Weissman et al. (2004) demonstrated that the DLPFC was active when subjects had to solve a cross-modal task with distraction. The DLPFC is located in the active areas of MS A and MS B, given their topography. Weissman et al. (2004) created two parts of the cross-model task, one with visual stimuli and auditory distraction and the other with auditory stimuli and visual distraction. When participants had to solve a visual task while listening to distracting auditory input, they found only activation of the left DLPFC for this type of task. Conversely, for an auditory task with visually distracting stimuli, no significant activation of the right DLPFC was found (Weissman et al., 2004). We expected for our paradigm to see stronger impact on MS B representing activity in the visual system in RS2. Nevertheless, we saw a decrease of MS parameters for MS A. The DLPFC is involved in directing attention to task-relevant stimuli (Weissman et al., 2004). Although we only evaluated the RS before and after the attention-demanding task, the DLPFC seems to be active. Therefore, the demands on DLPFC processes appear to be higher when attention is directed towards a visual process with a concurrent auditory distracting component (Weissman et al., 2004). More plausible seems to be the interpretation of Britz et al. (2010), who associated MS A with the auditory network (Britz et al., 2010). Thus, the decrease in duration, occurrence and contribution during RS2 could also be due to fatigue or adaptation of this network after auditory distraction, in particular, since these effects were not observed in RS2 of the C.C. in which only informative sound was provided.

### 4.2. MS C and the Cognitive Control Network

As several studies reported, MS C is associated with parts of the cognitive control network (Seeley et al., 2007; Britz et al., 2010; Custo et al., 2017). Britz et al. (2010) associated MS C with a fMRI RS network, named salience network (Britz et al., 2010). The salience network provides functions such as attention, planning or working memory in order to enable appropriate behaviour or achieve a specific goal (Breukelaar et al., 2017). In contrast to Britz et al. (2010) and Custo et al. (2017) estimated sources of the MS topographies with topographic electrophysiological state source-imaging (Britz et al., 2010; Custo et al., 2017). They determined seven mean MS patterns in their dataset, where MS C was split into two spatially correlated MS topographies (MS C and MS F) (Custo et al., 2017). Generators of MS C localised in the PCC and the precuneus, whereas MS F was generated in the dorsal ACC extending to the superior frontal gyrus, the bilateral middle frontal gyrus and bilateral insula. They identified these two MSs as part of the intrinsic default mode network, which deals with various aspects of self-related information (self-related evaluations, voluntary actions, episodic memory and planning) (Custo et al., 2017). For an extensive review, see Salomon et al. (2014).

After RS2 recording, no significant differences were found between C.C. and E.C. groups in questionnaire performance. However, brain areas and networks relevant for task solving were also associated with MS C and were activated after auditory distraction (Niendam et al., 2012; Breukelaar et al., 2017). According to our results, this seems to have taken up more resources for the video with auditory distraction than for the C.C.. The topography of MS C also supports this finding, as fMRI studies have found that cognitive control is induced by simultaneous activation of frontal and parietal cortex. Involved are the dorsal anterior cingulate cortex, the dorsolateral prefrontal cortex and the dorsal/posterior parietal cortex (Niendam et al., 2012; Breukelaar et al., 2017). Britz et al. (2010) found a relation between MS C and positive BOLD activations in the posterior part of the bilateral ACC, the bilateral inferior frontal gyri, the right anterior insula and the left claustrum (Britz et al., 2010). Other authors assume that MS C is associated with a portion of the anterior default mode network and not with the salience network (Custo et al., 2017; Seitzman et al., 2017; Michel and Koenig, 2018). Custo et al. (2017) suggested that the salience network described by Britz et al. (2010) rather corresponds to their MS F (Britz et al., 2010; Custo et al., 2017). They suggested that if only four microstate maps were used for the clustering procedure, MS C should be seen as a combination of the topographic patterns C and F, including the anterior and posterior cingulate cortices (Custo et al., 2017).

### 4.3. Transition Rates Show Interaction Between Salience and Attention Network After E.C.

Comparing the two transition rates in Figures 3B,C (original/delta), we noticed that the transitions in the original rate (left column) were dominated by transitions between MS A and MS B, or MS C and MS D. In the delta transition rate (right column), we observed differences of transitions from MS D and MS C to MS A in the E.C. and from MS A to MS C in comparison of the C.C. and E.C.. This suggests that the two transition rates should be considered separately. The delta transition rate was being calculated in combination with the theoretical transition model of Lehmann et al. (2005) and is independent of contribution and occurrence of the respective MSs (Lehmann et al., 2005).

As described above, the original transition rate decreases in RS2 for the transitions between MS A and MS B after auditory distraction (Figure 3B, left plot, blue arrows). Previous studies associated MS A with activity in the auditory and MS B with activity in the visual network (Britz et al., 2010; Custo et al., 2017; Seitzman et al., 2017). This suggests that the transition decrease between MS A and MS B (Figure 3B, left plot, blue arrows) reflects that auditory distraction override visual input. However, this interpretation is in contrast to the results of the other MS parameters. Here, a lower duration, occurrence and contribution for MS A can be seen. The original transition rate is influenced by the occurrences and contribution of the MSs and reflects the decrease of these parameters and this may also be an indication of fatigue in the auditory network.

A significant increase in the original transition rate in RS2 was also found for the transitions between MS C and MS D after auditory distraction (Figure 3B, left plot, red arrows). As mentioned above, these MSs are associated with the salience (MS C) and attention network (MS D) (Britz et al., 2010; Seitzman et al., 2017). Britz et al. (2010) found a correlation between MS D topography and ventral fronto-parietal areas being involved in switching and focusing of attention (Corbetta and Shulman, 2002; Britz et al., 2010). Thus, the increased transitions between these two MSs could reflect alternating activation of these two resting state networks, which corresponds to our hypotheses. As mentioned above Custo et al. (2017) split MS C in two seperate topographies, where only one corresponds to the salience network (Britz et al., 2010; Custo et al., 2017). Mishra et al. (2020) found that transitions between MSs are continuous and do not necessarily represent discrete transitions between MSs. Therefore, they suggested MS C as an interim state and not a main MS pattern (Mishra et al., 2020). The assumption of both studies relates to our results, as it argues for a close interaction between MS C and MS D.

The delta transition rate shows significant decreases (right column in Figure 3B) between the transitions from MS C to MS A and from MS D to MS A (vertical and oblique arrows, respectively). Some studies associated MS D with the dorsal attention network (Britz et al., 2010; Custo et al., 2017; Seitzman et al., 2017). This network is relevant for detecting behaviourally relevant stimuli. The topography of MS D includes more dorsal areas in the fronto-parietal cortex which are involved in switching and reorientation of attention (Corbetta and Shulman, 2002; Britz et al., 2010). MS C is associated with the salience network or the intrinsic default mode network (Britz et al., 2010; Custo et al., 2017). The decrease in the transition rate to the auditory network (MS A) and the decrease of MS parameters duration, contribution and occurrence of MS A, indicate that auditory influences are actively limited by the salience/ intrinsic default mode network (MS C) and attention network (MS D).

### 4.4. Transition Rates Are Influenced by the Salience and Attention Networks

The influence of the audiovisual paradigm on RS activity can be unravelled when comparing E.C. and C.C.. The results of the RS recordings were subtracted from each other to obtain a RS independent comparison between the two conditions. Transitions between MS A and MS B show significant decreases in the original transition rate after auditory distraction (Figure 3C, left plot, blue arrows). This suggests that the auditory distraction negatively affects the transitions between the two MSs. In contrast to the RS following the auditory distraction, only the transitions from MS C to MS D are significantly increased for the original transition rate in the comparison between E.C. and C.C. (Figure 3C, left plot, red arrows). Thus, the attention network seems to be more active, suggesting that the transitions between MS A and MS B are actively influenced by auditory distraction.

Comparison of the two conditions revealed significantly higher transition rates from MS A to MS C, using the delta transition rate (Figure 3C, right plot, red arrow). These result again suggest involvement of the salience network: we proposed that the salience network most likely actively filters out auditory distraction. This interpretation is also more plausible with regard to the other MS parameters, as the occurrence and contribution show an increase for MS C, while MS A showed a decrease in contribution (Figure 2I).

In summary, the salience, attention and intrinsic default mode networks are activated by our paradigm and appear to compensate for auditory distraction in order to protect memory content for the successful completion of the questionnaire. We would rather not assume that those networks are actively blocking any further influence from new sensory input in favour of memory maintenance, as no significant differences in questionaire performance were seen between C.C. and E.C.. This is also underlined by the comparison between RS1 and RS2 in the C.C., which showed no significant differences in terms of MS parameters.

## 5. Conclusion

Distracting music during active audiovisual perceptions had a significant impact on microstates. In particular we observed effects on MS A and MS C, reflecting activity changes in the auditory system and in the salience or intrinsic default mode network. The control group expressed hardly any changes in MS parameters as a consequence of watching and memorising the video. The experimental group, being exposed to auditory distraction, showed effects on both MS A and MS C, the latter effects being more pronounced. The uniformity of response among subjects in the experimental groups is a strong indication that these measurements are reliable. The most plausible interpretation is that brain activity related to auditory system activity (MS A) is fatigued by auditory distraction. Furthermore, the salience network and the attention network are active in order to memorise the answers for the following questionnaire and to minimise the influences of the distracting background music. Increased activity of the salience network combined with changes in transition rates between MS C and MS A and between MS D and MS A suggests active suppression of auditory distraction. Thus, MS analysis can provide important information about the cerebral processes that occur in everyday life.

## Data Availability Statement

The datasets presented in this article are not readily available as they are being used for further analysis. Requests to access the datasets should be directed to Matthias H. J. Munk, matthias.munk@uni-tuebingen.de, Ute Korn, ute.korn@bio.tu-darmstadt.de.

## Ethics Statement

Ethical review and approval was not required for the study on human participants in accordance with the local legislation and institutional requirements. The patients/participants provided their written informed consent to participate in this study.

## Author Contributions

FH and MM designed research. FH, UK, MM, and RG acquired the data. MK, KH, SA, RS, and HJ provided hardware/software support for data acquisition and analysis. UK and MK analysed the data. KH provided support for statistical analysis. UK prepared the first draft of the manuscript. MK, KH, RG, and MM corrected and improved the draft. All authors have read, commented, and approved the final version of the manuscript prior to submission.

## Funding

UK was funded by the Hubert Markl-scholarship of the Carlo and Karin Giersch Foundation at Technische Universität Darmstadt.

## Conflict of Interest

The authors declare that the research was conducted in the absence of any commercial or financial relationships that could be construed as a potential conflict of interest.

## Publisher's Note

All claims expressed in this article are solely those of the authors and do not necessarily represent those of their affiliated organizations, or those of the publisher, the editors and the reviewers. Any product that may be evaluated in this article, or claim that may be made by its manufacturer, is not guaranteed or endorsed by the publisher.

## Footnotes

^1^ http://www.thomaskoenig.ch/index.php/software/microstates-in-eeglab
