# Supplementary material for: EEG-Microstates Reflect Auditory Distraction After Attentive Audiovisual Perception Recruitment of Cognitive Control Networks
Source: Front Syst Neurosci. 2021 Dec 9;15:751226. doi: 10.3389/fnsys.2021.751226 (PMC8696261; doi:10.3389/fnsys.2021.751226)
Supplement: Supplementary file 1 [file Data_Sheet_1.PDF]

Supplementary Material

1 SUPPLEMENTARY DATA

1.1 Method: Questionaire

Questions about the TV report: Please answer all questions as quickly as possible! This should not take longer than 5 minutes!

1. Have you seen the film before?
   ☐ Yes ☐ No

2. How did you like the film?
   Not at all Very Good
   ☐ 1 ☐2 ☐3 ☐4 ☐5 ☐6 ☐7 ☐8 ☐9 ☐10

3. Is there anything in the contribution that disturbed you?
   Answer: ______________________________

4. How many facts and figures do you estimate you got?
   None All
   ☐ 1 ☐2 ☐3 ☐4 ☐5 ☐6 ☐7 ☐8 ☐9 ☐10

5. What was the amount in euros of the losses caused by the 2008 financial crisis?
   ______________ € ☐ Does not emerge from the film

6. How many signatures did the street preacher collect for his petition in 2011?
   ☐ 100
   ☐ 1000
   ☐ 10,000 ☐ Does not emerge from the film

7. Cloze:
   _____ % of the total world population have more assets than _____ % of the world population

8. At which university are tuition fees $50,000 per year?
   ☐ Stanford University
   ☐ Yale University
   ☐ Harvard University

9. What colour is the dress of the girl in the "private cinema"?
   ☐ red
   ☐ yellow
   ☐ blue ☐ Does not emerge from the film

10. How much salary does a Kenyan get on average per month?
    ☐ 50€
    ☐ 70€

☐ 100€

11. Which three measures/events could close the gap between rich and poor again?
Answer: ______________________________

12. Which countries are covered in the article?
☐ United Arab Emirates ☐ Thailand ☐ Singapore ☐ USA
☐ Kenya ☐ South Africa ☐ Poland ☐ Russia ☐ Spain

1.2 Method: MS Analysis

Here, k-means clustering was used to segment the classes of map topographies. For this study, the number of clusters was defined as k = 4. Those maps were estimated solely at GFP peaks. A permutation algorithm was used, which maximizes the common variance across subjects to computed mean classes across participants (Lehmann et al., 1998). In order to determine MS, we used mean MS classes across all participants and conditions as template maps. Classes of MS topographies were averaged for each subject separately and then averaged across all subjects per group and state (Koenig et al., 2002). The topographic maps were then fitted back to the data post hoc and compared to template maps (Michel and Koenig, 2018; Krylova et al., 2021). The EEG topography of each individual participant was assigned to one of these four known MS topographies, based on the maximal absolute value of the Pearson correlation coefficient (Michel and Koenig, 2018). Temporal sequences of maps with identical topography were classified as coherent MS. Time points between two MS were assigned to the temporally closest MS class, and time points before the first and after the last GFP peak in each epoch were rejected (Krylova et al., 2021).

1.3 Method: Statistical Analysis

To test for equal medians between C.C. and E.C., the two-sided Wilcoxon rank sum test was used (function *ranksum*), which is identical to the Mann-Whitney U test (MWU). Here, the null hypothesis (H_0) is specified as two independent samples are coming from distributions with equal medians. The two sets of data are assumed to come from continuous distributions that are identical except possibly for a location shift, but are otherwise arbitrary (Hollander and Douglas, 1999; Gibbons and Chakraborti, 2010). Wilcoxon signed rank tests (function *signrank*) were conducted for testing RS1 and RS2 within each separate experimental condition. Here, the H_0 is specified as the difference between the matched samples in RS1 and RS2 coming from a distribution whose median is zero, and is tested via this paired two-sided test. The differences RS1-RS2 are assumed to come from a continuous distribution, symmetric about its median. The two-sided p-value is computed by doubling the most significant one-sided value. The signrank function calculates the p-value using the z-statistic, given by

$$z = \frac{(W - n(n+1)/4)}{\sqrt{\frac{n(n+1)(2n+1) - \text{tieadj}}{24}}} \tag{S1}$$

where n is the sample size of the difference RS2 – RS1 and W is the sum of the ranks of positive differences between the observations in the two samples. For the two-sample case, the tie adjustment value (*tieadj*) was used (Hollander and Douglas, 1999; Gibbons and Chakraborti, 2010).

1.4 Results: Changes in MS Topography

After clustering and reordering steps, the final topographies of all MS were estimated. The averaged topographies per condition and resting state recording were analysed. Comparing the topographies of MS B in the two RS recordings of the control condition, differences in topographies are recognizable. The paradigm per se, without auditory distraction, had an impact on the topography of MS B: in RS2, the anterior pattern was shifted more to the left, while during RS1 the pattern was more frontally distributed. MS C also showed differences between the two RS recordings: in RS1, the pattern was more nasal in the frontal head region, while in RS2 it extended more into the frontal head region.

1.5 Discussion: Differences in MS Topography justified by the Methods

MS topographies differ across the conditions and within the first RS recording (Figure 1). The small differences between the topographies of the RS1 measurements may be due to the EEG method. The EEG method has very poor spatial resolution and an EEG electrode not only receives signals from the directly underlying neural structures, but also from the entire brain by volume conductance. This had an influence on the *k*-means cluster algorithm (Cohen, 2014). *k*-means clustering produces slightly different results with each new initialization. It should be noted that this could affect the choice of topography, which was selected according to similarity to the commonly accepted MS topographies. The explained variance of all data after clustering reached $79.1\% \pm 3.8\%$ for RS1 and $80.2\% \pm 3.1\%$ for RS2 in both experimental conditions. The four selected clusters therefore explain a substantial amount of the overall variance of our data which is sufficient to evaluate MS.

2 SUPPLEMENTARY TABLES AND FIGURES

2.1 Tables

Table S1. Sampling Rates of EEG Measurements.

No. Subjects	Year	Sampling Rate [Hz]
11	2017	500
10	2018	500
6	2019	250

2.2 Figures

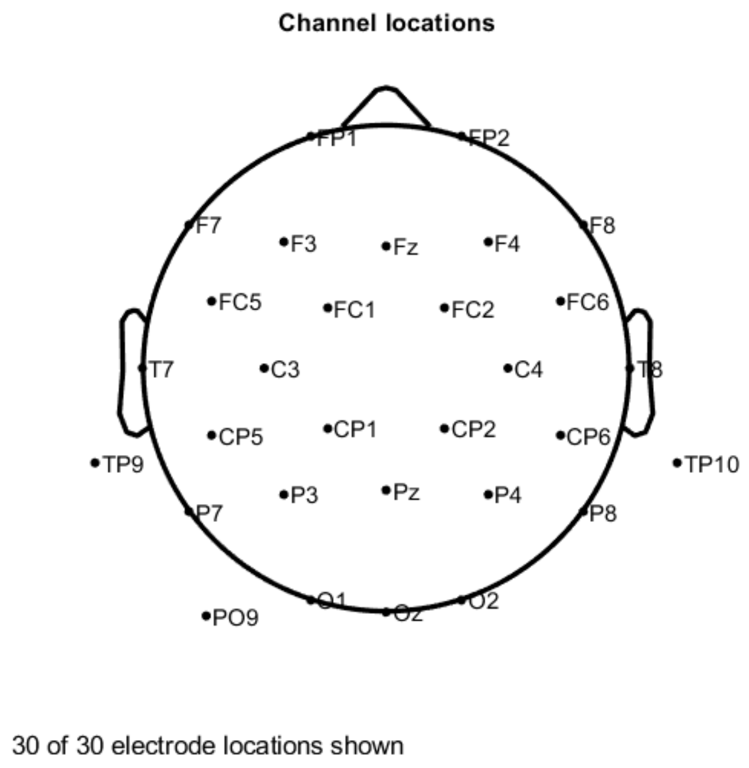

Figure S1. Topographical plot shows the 30 channel locations (10-20-system) which are selected for final analysis. Left side is left, right side is right and frontal is up. This figure was made by the MATLAB toolbox EEGLAB. Electrode PO9 is positioned in the occipital head areas, whereas the electrodes TP9 and TP10 are positioned at the left and right mastoid.

REFERENCES

Cohen, M. X. (2014). *Analyzing Neural Time Series Data: Theory and practice* (Cambridge, Massachusetts: MIT Press)

Gibbons, J. D. and Chakraborti, S. (2010). *Nonparametric Statistical Inference* (Chapman and Hall/CRC). doi:10.1201/9781439896129

Hollander, M. and Douglas, W. A. (1999). *Nonparametric Statistical Methods* (Wiley-Interscience)

Koenig, T., Prichep, L., Lehmann, D., Sosa, P. V., Braeker, E., Kleinlogel, H., et al. (2002). Millisecond by millisecond, year by year: Normative eeg microstates and developmental stages. *NeuroImage* 16, 41–48. doi:10.1006/nimg.2002.1070

Krylova, M., Alizadeh, S., Izyurov, I., Teckentrup, V., Chang, C., van der Meer, J., et al. (2021). Evidence for modulation of eeg microstate sequence by vigilance level. *NeuroImage* 224, 117393. doi:10.1016/j.neuroimage.2020.117393

Lehmann, D., Strik, W. K., Henggeler, B., Koenig, T., and Koukkou, M. (1998). Brain electric microstates and momentary conscious mind states as building blocks of spontaneous thinking: I. visual imagery and abstract thoughts. *International Journal of Psychophysiology* 29, 1–11. doi:10.1016/S0167-8760(97)00098-6

Michel, C. M. and Koenig, T. (2018). Eeg microstates as a tool for studying the temporal dynamics of whole-brain neuronal networks: A review. *NeuroImage* 180, 577–593. doi:https://doi.org/10.1016/j.neuroimage.2017.11.062
